# Efficiency Enhanced Grating Coupler for Perfectly Vertical Fiber-to-Chip Coupling

**DOI:** 10.3390/ma13122681

**Published:** 2020-06-12

**Authors:** Zan Zhang, Xiaotao Shan, Beiju Huang, Zanyun Zhang, Chuantong Cheng, Bing Bai, Tianxi Gao, Xiaobo Xu, Lin Zhang, Hongda Chen

**Affiliations:** 1 School of Electronic and Control Engineering, Chang’an University, Xi’an 710064, China; 2016903901@chd.edu.cn (X.S.); baibing@chd.edu.cn (B.B.); tianxigao@chd.edu.cn (T.G.); xuxiaobo@chd.edu.cn (X.X.); zhanglin_dk@chd.edu.cn (L.Z.); 2 Chinese Academy of Sciences, Beijing 100083, China; bjhuang@semi.ac.cn (B.H.); chengchuantong@semi.ac.cn (C.C.); hdchen@semi.ac.cn (H.C.); 3 School of Electronics and Information Engineering, Tianjin Polytechnic University, Tianjin 300387, China; zhangzanyun@tjpu.edu.cn

**Keywords:** silicon photonics, grating coupler, backside metal mirror, photonic integrated circuit

## Abstract

In this work, a bidirectional grating coupler for perfectly vertical coupling is proposed. The coupling efficiency is enhanced using a silicon nitride (Si_3_N_4_) layer above a uniform grating. In the presence of Si_3_N_4_ layer, the back-reflected optical power into the fiber is diminished and coupling into the waveguide is increased. Genetic algorithm (GA) is used to optimize the grating and Si_3_N_4_ layer simultaneously. The optimal design obtained from GA shows that the average in-plane coupling efficiency is enhanced from about 57.5% (−2.5 dB) to 68.5% (−1.65 dB), meanwhile the average back-reflection in the C band is reduced from 17.6% (−7.5 dB) to 7.4% (−11.3 dB). With the help of a backside metal mirror, the average coupling efficiency and peak coupling efficiency are further increased to 87% (−0.6 dB) and 89.4% (−0.49 dB). The minimum feature size of the designed device is 266 nm, which makes our design easy to fabricate through 193 nm deep-UV lithography and lowers the fabrication cost. In addition, the coupler proposed here shows a wide-band character with a 1-dB bandwidth of 64 nm and 3-dB bandwidth of 96 nm. Such a grating coupler design can provide an efficient and cost-effective solution for vertical fiber-to-chip optical coupling of a Wavelength Division Multiplexing (WDM) application.

## 1. Introduction

Silicon photonics has enabled a wide range of applications ranging from light-based communication to interconnect to low-cost lab-on-a-chip systems [1]. Benefitting from the mature complementary metal-oxide-semiconductor (CMOS) technology, various photonic devices have been demonstrated base on low loss silicon-on-insulator (SOI) waveguide, showing great promise for electronic-photonic integrated circuits, high-density photonic integrated circuits (PICs), and three-dimensional (3D) photonic integration [2,3,4,5,6]. However, since there is no mature silicon-based light source, a fiber coupler is still needed to couple off-chip light from a single mode fiber (SMF) into a submicron SOI waveguide in a PIC [7]. Inverse tapers and grating couplers are the two most commonly used interfaces between a silicon PIC and SMF. High coupling efficiency (CE) and wide bandwidth can be achieved simultaneously using inverse tapers [8]. However, there are a number of drawbacks using such approaches. Typically, specialized optical fibers and cleaved devices with polished facets are required. Very precise optical alignment is needed, which drastically increases the cost. The placement of inverse tapers are quite inconvenient since they have to couple light from the edge of the PIC chip. Grating couplers have been studied extensively and are attractive because they are easier to fabricate, are more flexible in their placement, and they enable to test their performance on the wafer-scale [9].

Conventional grating couplers couple light from tilted fiber to avoid second-order reflection [10,11]. Fiber angle tuning and polishing are always needed in optical tests and packaging of silicon PICs, which are disadvantageous for rapid wafer-scale tests and low-cost photonic packaging [12,13]. Therefore, perfectly vertical grating couplers with high CE would be very attractive as they can further lower the cost of photonic packaging.

Many designs have been proposed to achieve perfectly vertical couplers, such as a slanted grating coupler, chirped grating coupler, a coupler with extra reflector, and dual-layer grating coupler. A slanted grating coupler breaks the symmetry problem associated with the surface normal coupling between fiber and the planar waveguide, resulting in higher radiation directionality [14]. Chirped grating is a common approach to achieve efficient vertical coupling because chirping the grating structure will introduce asymmetry to the grating coupler [15,16]. Another way to introduce asymmetry to the grating is to add an extra etched reflector [17,18]. With the help of the reflector, coupling into unwanted modes could be decreased. As for a dual-layer grating coupler, an inter-layer lateral shift is introduced between the two layers to mimic tilted mirror behavior [19,20,21,22], so that high directionality and low back-reflection could be simultaneously realized. However, these approaches require either extra fabrication processes or sophisticated device structure with quite small minimum feature size, which increases the fabrication cost drastically and affects the fabrication error tolerance.

In order to avoid complicated design or extra fabrication steps, a grating coupler that enables vertical to in-plane bidirectional transmission with bi-layer anti-reflection cladding was proposed in our previous work [23]. The bidirectional grating coupler functions as both a fiber coupler and optical power splitter, so that it cannot only act as a highly efficient vertical coupler but also work in Mach–Zehnder type optical modulators [24,25,26]. However, directly depositing Si_3_N_4_ on SOI grating introduces extra deposition and etching steps into CMOS technology. In this work, we propose a new design of a bidirectional grating coupler with enhanced efficiency. Perfectly vertical coupling between SMF and SOI waveguide with high efficiency is numerically demonstrated. With the help of an additional Si_3_N_4_ layer above grating, enhanced CE is achieved with a cost-effective uniform grating coupler. Since the grating is covered by silicon oxide (SiO_2_), a Si_3_N_4_/SiO_2_ interface is created above the grating. With the help of this interface, maximal coupling into the guided mode and destructive interference in unwanted modes can be achieved. The reflection back into the fiber is reduced and total in-plane CE is increased. Compared with our previous work [23], both Si_3_N_4_/SiO_2_ structures are above the grating to reduce the back-reflection in both designs, but in a different manner. In our previous work, the Si_3_N_4_ layer was sandwiched by the grating and silicon oxide, while in this new design, the Si_3_N_4_ layer is above the silicon oxide. The change of the order makes the proposed device more fabrication-compatible with CMOS technology, since Si_3_N_4_ is a commonly used dielectric as the passivation layer in back end of line in the CMOS process. We believe that such a design can provide an efficient and cost-effective solution for vertical coupling applications and low-cost fiber packaging for silicon PICs.

## 2. Device Structure and Principle

**Figure 1:**
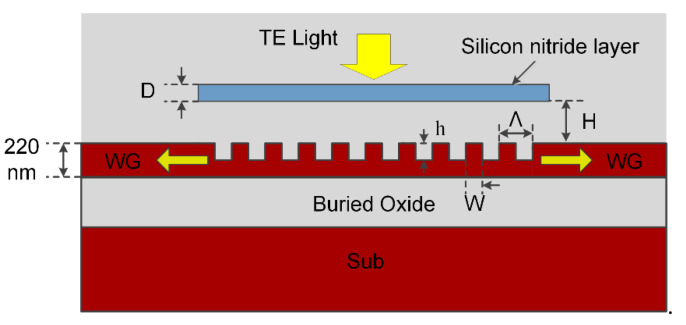
Schematic diagram of the device configuration.

A schematic of the vertical grating coupler is shown in Figure 1. As can be seen clearly, the device we propose is based on a uniform grating with two opposite in-plane transmission ports. The core structure of the device is a uniform grating with period of Λ. Compared with some sophisticated designs, such as chirped grating couplers [18,22], uniform grating couplers have a fairly larger minimum feature size about half of the wavelength in the SOI waveguide. This larger minimum feature size ensures that the proposed coupler is a cost-effective device.

When a perfectly vertical SMF is placed in the center of the uniform grating, transversal electric (TE) polarized light from the fiber is diffracted by the grating structure and coupled into two waveguide modes with opposite directions. According to the Bragg condition, the perfectly vertical coupling can be achieved with a carefully designed grating parameter. The total CE for perfectly vertical in-plane coupling is mainly affected by up-reflection and substrate leakage. The up-reflection power attracts more concern since it will not only decrease the CE but also lead to light source degradation because of the power reflected back through fiber.

In the current design we add a specific Si_3_N_4_ layer with carefully designed thickness *D* and gap to grating *H* to reduce the up-reflection power. By introducing the Si_3_N_4_ layer, a Si_3_N_4_/SiO_2_ interface is created above the grating. Considering the silicon/SiO_2_ interface introduced by grating itself, the whole structure can be seen as a Fabry–Pérot interferometer. Thus, the reduction of up-reflection can be explained with the help of the Fabry–Pérot interferometer; constructive interference occurred in guide mode and destructive interference in up-reflected mode.

## 3. Design and Simulation

### 3.1. Design of a Vertical Grating Coupler with Si_3_N_4_ Layer

We started with the design of a vertical grating coupler without an additional Si_3_N_4_ layer. The coupler is based on a uniform grating where the first-order diffraction couples light out of the waveguide, producing a surface-normal propagating field [27]. According to the Bragg condition, we can achieve a 90° coupling with a grating when the grating period Λ equals the wavelength divided by the effective refractive index *n*_eff_. The goal of the simulation and optimization is to obtain the highest average CE in C band (1530~1565 nm). The whole simulation was done using two-dimensional finite-difference time-domain (FDTD) simulations with commercial software Lumerical FDTD Solutions (Lumerical Inc., Vancouver, Canada). The device was designed to be fabricated on a SOI substrate with 220-nm-thick top silicon and 2-μm-thick buried oxide. The grating groove depth *h* was chosen as 70 nm, a measurement widely used in most silicon photonic fabrication platforms. The grating period number was 17 and the refractive index of Si_3_N_4_ was 2.03 [28].

**Figure 2:**
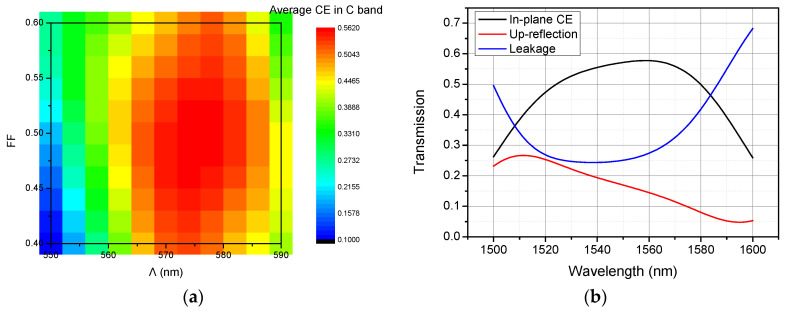
(**a**) The average coupling efficiency (CE) in C band with different Λ and FF. The grating coupler with Λ = 575 nm and filling factor (FF) = 0.49 achieves an average CE in C band of about 56.3%. (**b**) Calculated transmission spectra of the simple vertical grating coupler with Λ = 575 nm and FF = 0.49.

Grating is a polarization sensitive device and TE polarized light is of great interest in most cases of silicon PICs. Therefore, the input SMF mode was modeled using a TE polarized Gaussian source with mode waist of 5.2 μm. The average CE in C band with different Λ and filling factor (FF = W/Λ, where W is the grating teeth width) are shown in Figure 2a, where Λ = 575 nm and FF = 0.49 were chosen as the optimal design parameters for the vertical grating coupler with an average CE in C band of about 56.3%. The up-reflection and substrate leakage of the simple vertical grating coupler using these optimal parameters was then calculated and plotted in Figure 2b. As shown clearly, nearly half of the input light power is either coupled to the substrate or reflected back to fiber. The average up-reflection power in C band is so high that the return loss is reaching −7.5 dB. High return loss will induce damage to the light source, which is unacceptable in a practical system.

**Figure 3:**
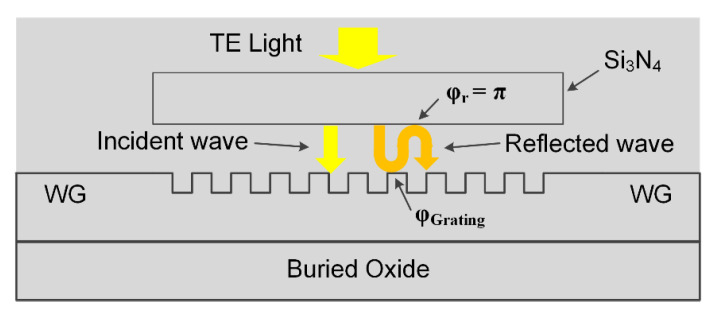
Schematic of the phase difference between incident wave and reflected wave.

To reduce the up-reflection, we then added an additional Si_3_N_4_ layer above the grating region. As discussed above, the Si_3_N_4_ layer introduced a Si_3_N_4_/SiO_2_ interface that plays a key role in the CE enhancement. The gap *H* between Si_3_N_4_ layer and grating has a major influence on the CE. It should be note that the incident wave will be partially reflected by the grating, which is the up-reflection wave. In the presence of Si_3_N_4_ layer, the up-reflected wave partially reflects back towards the grating at the Si_3_N_4_/SiO_2_ interface, as illustrated in Figure 3. The gap *H* should be chosen such that the waves reflected back by the Si_3_N_4_/SiO_2_ interface interfere constructively with the wave coupled into the grating. To ensure the constructive interference, the reflected wave and incident wave should be in phase. As depicted in Figure 3, the phase difference between the incident wave and reflected wave is:

(1) $$\Delta \varphi =2\pi \frac{2H}{\lambda_{0}/n_{OX}}+\varphi_{r}+\varphi_{Grating}.$$

where $\lambda_{0}$ is the wavelength of the input light, $n_{OX}$ is the refractive index of SiO_2_, $\varphi_{r}$ is the phase shift at the Si_3_N_4_/SiO_2_ interface, which is *π*, and $\varphi_{Grating}$ is the phase shift at the grating/SiO_2_ interface. When Δ*φ* equals 2*m*π (*m* is an integer), constructive interference occurs and hence coupling into the grating will be enhanced.

**Figure 4:**
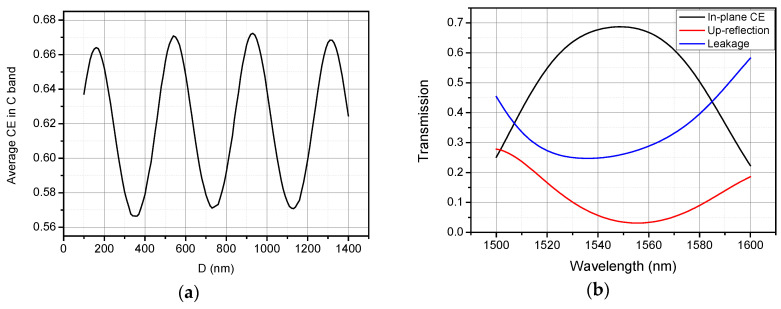
(**a**) Average CE in C band with different *D*; (**b**) calculated transmission spectra of vertical grating coupler with *H* = 530 nm and *D* = 930 nm.

For simplicity, we assume that $\varphi_{Grating}$ = *π*, since the effective refractive index of the grating is higher than $n_{OX}$. Therefore, constructive interference occurs when $H=m\lambda_{0}/\left(2n_{OX}\right)$. Considering *λ*_0_ = 1550 nm, $n_{OX}$ is 1.45, and *m* is 1, then the gap *H* is calculated as 530 nm. With this calculated gap, the thickness of the Si_3_N_4_ layer *D* is determined by a parameter sweep through a series of numerical simulations, as shown in Figure 4a. The parameters of the grating are the same as the vertical grating coupler designed above. As shown in Figure 4a, the CE is a periodical function of the thickness *D*. By choosing *D* properly to add destructive interference between the wave reflected by grating and the wave reflected by Si_3_N_4_ layer, the back-reflection is diminished so that the coupling into guided mode is enhanced. From Figure 4a, *D* is set to be 930 nm to achieve the highest average CE in C band of about 67.2%; the waves are reflected by grating and reflected by Si_3_N_4_ interfere at the upper Si_3_N_4_/SiO_2_ interface. The up-reflection and substrate leakage of the efficiency enhanced vertical grating coupler with *H* = 530 nm and *D* = 930 nm are then calculated and plotted in Figure 4b. As shown clearly, the peak CE is about 68.7% (−1.63 dB) at 1549 nm, and 1-dB and 3-dB bandwidths are about 56 nm and 85 nm, showing a wideband feature. The average up-reflection of the efficiency enhanced coupler is as low as 4.9%, showing 5.6 dB improvement in return loss compared to the simple vertical grating coupler without Si_3_N_4_ layer. However, as shown in the blue curve in Figure 4b, around 25% of the light power leak into the silicon substrate, which limits the total in-plane CE.

**Figure 5:**
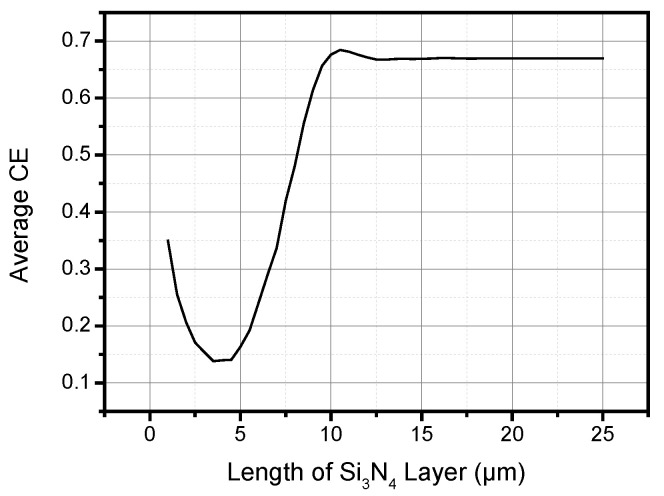
Average in-plane CE in C band with different lengths of Si_3_N_4_ layer. As long as the Si_3_N_4_ layer is longer than the grating, the coupling efficiency will not vary with the length of the Si_3_N_4_ layer.

The effect of length of the Si_3_N_4_ layer on CE is analyzed through a parameter sweep. Figure 5 shows that the length of the Si_3_N_4_ layer will only affect the coupling seriously if it is smaller than the length of grating. There is a peak CE when the length is about the same as the grating length shown in Figure 5. However, as long as the Si_3_N_4_ layer is longer than the grating, the coupling efficiency will not vary with the length of the Si_3_N_4_ layer. The peak CE is believed to be the result of edge diffraction of the Si_3_N_4_ layer. However, the peak CE is about only 0.015 higher than the CE when the Si_3_N_4_ layer is longer than the grating. Therefore, it is possible to deposit a full Si_3_N_4_ layer to avoid an extra etching process. Directly using the Si_3_N_4_ as the passivation layer over the SiO_2_ layer is also possible since the UV glue or index matching fluid will be used between the fiber and the photonic chip in most cases.

**Figure 6:**
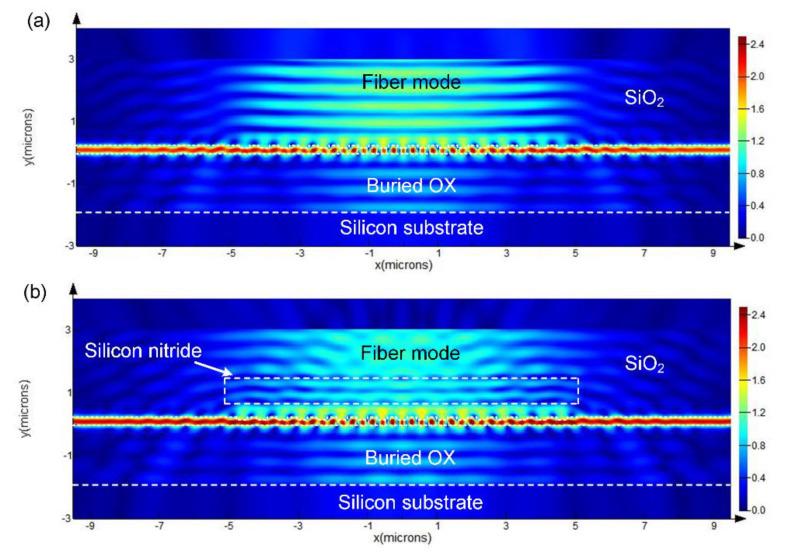
The cross-sectional view of the electric intensity profiles at wavelength of 1550 nm for (**a**) vertical grating coupler without Si_3_N_4_ layer and (**b**) CE enhanced vertical grating coupler with *H* = 530 nm and *D* = 930 nm.

The cross-sectional view of the electric intensity profiles at wavelength of 1550 nm was calculated for grating coupler without and with an additional Si_3_N_4_ layer respectively, as shown in Figure 6. From these result plots, we can clearly see the coupling scheme and the power flow in different directions. Obviously, constructive interference occurred between the Si_3_N_4_ layer and grating, resulting in diminished up-reflection.

### 3.2. Optimization Using Genetic Algorithm (GA)

As shown in Figure 1, there are five layers in the whole device, namely, silicon substrate, buried oxide, top silicon, covered SiO_2_ and Si_3_N_4_; this results in five interfaces existing in the whole coupler. Apparently, all interfaces could have contributed to the reduction of up-reflection. Due to the phase response of the sub-wavelength grating and the effect of all interfaces, the phase-matching condition is a little complicated, and accurate analytical calculation of the thickness *D* and gap *H* is quite difficult. Moreover, when considering the grating and Si_3_N_4_ layer as a whole device, the optimal coupling condition may happen with different Λ, FF, and *h* compared to the vertical grating coupler designed above. Therefore, in order to achieve good coupling performance, all the five parameters (*D*, *H*, Λ, FF and *h*) should be carefully designed, which is a typical multi-peak problem that needs a lot of calculation.

Fortunately, the two-dimensional FDTD simulation of the proposed device is not time-consuming, so that we can turn to GA to help find the optimal design parameter. GA is a global search algorithm which applies the principle of survival of the fittest. It does not depend on the gradient information when optimizing the calculation and does not require the objective function to be continuous and steerable, making it suitable to optimize the parameters of a grating coupler. Moreover, it has already proven helpful in related fields [11,16,29,30].

**Figure 7:**
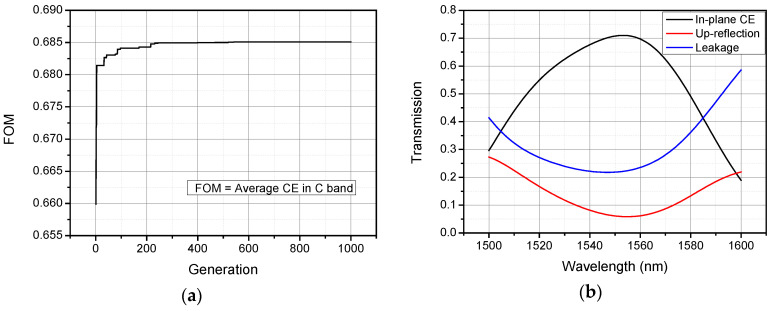
Results for genetic algorithm (GA)-optimized coupler (**a**) runtime statistics of maximum and average CE; (**b**) calculated transmission spectra for the final optimal design with Λ = 579 nm, FF = 0.46, *D* = 959 nm, *H* = 498 nm, and *h* = 75 nm.

Here we set the average CE in C band as the figure of merit (FOM). Simulation results of the final optimized design obtained through GA are shown in Figure 6. The FOM trend with the iterative process is shown in Figure 7a. As can be seen, the simulation reaches convergence after the 600th generation. The optimal parameter of the whole coupler is Λ = 579 nm, FF = 0.46, *D* = 959 nm, *H* = 498 nm, and *h* = 75 nm. The GA-optimized coupler is capable of achieving average CE in C band of 68.5% and the peak CE of 71% occurred in 1553 nm with a 1-dB bandwidth of 53 nm and 3-dB bandwidth of 85 nm, as shown in the transmission spectra. Compared with the coupler designed in Section 3.1., the average up-reflection in C band of the GA-optimized coupler is increased slightly to 7.4% as shown in Figure 7b.

Obviously, the optimal *H* and *D* obtained through GA are a little different from the calculated *H* and *D* in Section 3.1. This difference occurs because of the simplification we made that $\varphi_{Grating}$ = *π*. Apparently, phase shift of the grating is more complicated because of effects of all interfaces in whole grating. However, the difference between the calculated CE in Section 3.1. and the GA-optimized design is quite small, which indicates that the proposed device is a fabrication-tolerant design. Moreover, the minimum line width of the proposed coupler is 266 nm, which can be easily fabricated with 193 nm deep-UV lithography and lowers the fabrication cost.

### 3.3. Combined Coupler and Backside Metal Mirror

**Figure 8:**
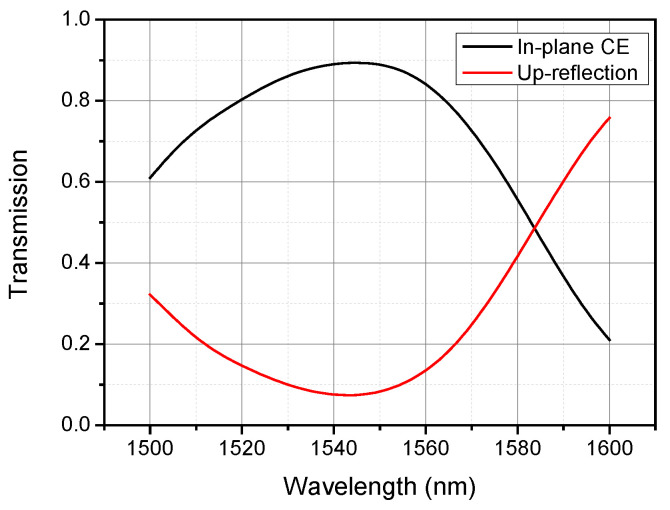
Calculated transmission spectra for the GA-optimized design with a 60-nm-thick aluminum backside metal mirror, showing increased CE and bandwidth.

To further increase the efficiency of grating couplers, the directionality of the grating should be optimized since there are still about more than 20% incident power couple into silicon substrate at 1550 nm in our design. A backside metal mirror has proven to be effective in redirecting the optical power diffracted to the substrate, leading to a high CE [31,32,33]. In addition, the backside metal mirror can be realized with CMOS compatible processes such as deep-UV photolithography, etching, and metal deposition, thus simplifying the integration of a metal mirror with grating coupler [33,34]. In our simulation, a 60-nm-thick aluminum layer was placed under the buried oxide layer to act as a backside metal mirror. As depicted in Figure 8, an average CE of 87% and peak CE of 89.4% were achieved, which show an appreciable improvement of around 1.05 dB in the average CE. Since the diffracted power penetrating the grating is reflected at the metal mirror, an important part is redirected to the grating and coupled into guided mode, resulting in enhanced efficiency of the grating coupler. Moreover, a 1-dB bandwidth amelioration from 53 to 64 nm and a 3-dB bandwidth amelioration from 85 to 96 nm are achievable.

## 4. Discussion

As mentioned in Section 3.1., the grating period of the proposed device is about *λ*_0_/*n*_eff_, and the second-order back-reflection is of great concern. In the case of coupling from chip to fiber, lights will be incident to the grating region from two waveguides on both sides of the proposed bidirectional grating. While for a conventional grating coupler connected to a single waveguide, light is incident from one waveguide. Therefore, the second-order back-reflection will be of great concern when the period of the conventional grating coupler is around *λ*_0_/*n*_eff_.

**Figure 9:**
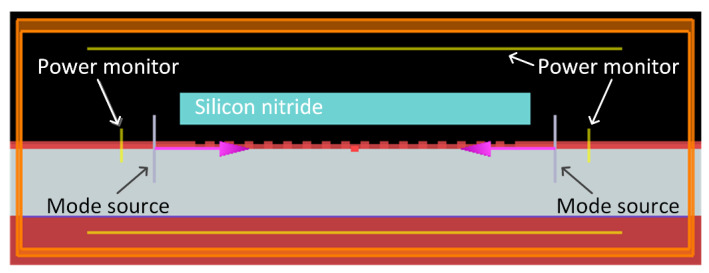
The simulation set-up in the case of chip-to-fiber coupling for the bidirectional grating. Power monitors are set behind mode sources on both sides of grating to calculate back-reflection.

**Figure 10:**
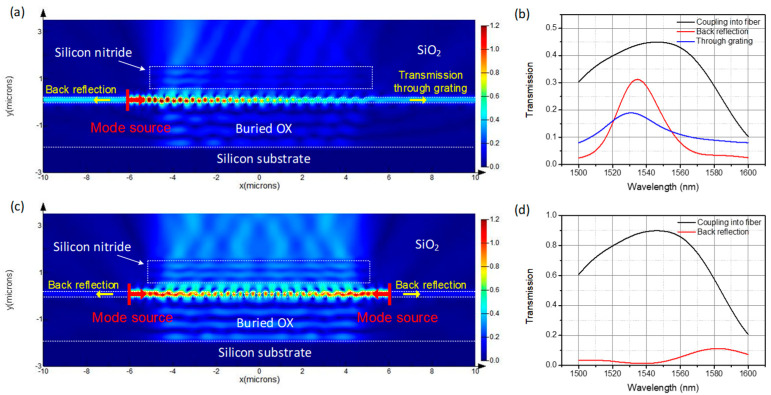
Two-dimensional (2D) finite-difference time-domain (FDTD) simulation results for grating coupler with one mode source positioned at left waveguide: (**a**) cross-sectional view of the electric intensity profile at wavelength of 1550 nm; (**b**) waveguide-to-fiber CE, second-order back-reflection into waveguide and transmission through grating. 2D FDTD simulation results for bidirectional grating coupler with two mode sources positioned on both sides: (**c**) cross-sectional view of the electric intensity profile at wavelength of 1550 nm; (**d**) waveguide-to-fiber CE and back-reflection into waveguide.

The back-reflection is calculated using power monitors positioned behind mode sources in the two-dimensional (2D) FDTD simulation. The CE into fiber is calculated by overlap integral between the fiber mode and the field from the power monitor over the grating. The simulation set-up with two mode sources is depicted in Figure 9, and all parameters of the grating are the same as the ones in Section 3.3. Firstly, simulation with one mode source positioned at the left waveguide is carried out to analyze the performance of a conventional grating coupler. As shown in Figure 10a,b, the back-reflection reaches as high as 31% at 1535 nm and about 18% power of light transmit through the grating. However, for the bidirectional grating, since there are guided waves propagating to the grating region from both sides, the wave reflected back into the right waveguide interferes destructively with the wave transmitting through the grating from the left waveguide. The same happens to the wave reflected into the left waveguide. Destructive interference between the reflected wave and transmitted wave results in diminished back-reflection into waveguides of about 1.5%; the simulation results are shown in Figure 10c,d. The CE from waveguides to fiber matches well with the case fiber-to-chip coupling.

## 5. Conclusions

In conclusion, a high efficiency and broadband vertical grating coupler is designed and numerically demonstrated. Assisted by a Si_3_N_4_ layer above the grating, the total in-plane CE of the vertical grating coupler is enhanced greatly while up-reflection is diminished. Genetic algorithm is employed to simultaneously optimize the grating and Si_3_N_4_ layer. The average CE and up-reflection in C band of GA-optimized coupler are 68.5% (−1.65 dB) and 7.4% (−11.3 dB), respectively. With the help of a backside metal mirror, the average CE in C band and peak CE are further increased to 87% (−0.6 dB) and 89.4% (−0.49 dB), respectively. The minimum feature size of the designed device is 266 nm, which makes our design easy to fabricate through 193 nm DUV lithography. With a large optical bandwidth (1-dB bandwidth of 64 nm, 3-dB bandwidth of 96 nm) and flat-top filtering characteristic, this vertical coupler is believed to be suitable for the optical interface of a WDM application and low-cost fiber packaging for silicon PICs.
